# Involvement of *Campylobacter* Species in Spotty Liver Disease-like Lesions in Broiler Chickens Detected at Meat Inspections in Miyazaki Prefecture, Japan

**DOI:** 10.3390/microorganisms12122442

**Published:** 2024-11-27

**Authors:** Piyarat Jiarpinitnun, Akira Iwakiri, Naoyuki Fuke, Pornsawan Pongsawat, Chizuru Miyanishi, Satomi Sasaki, Takako Taniguchi, Yuto Matsui, Taradon Luangtongkum, Kentaro Yamada, Naoaki Misawa

**Affiliations:** 1 Laboratory of Veterinary Public Health, Graduate School of Medicine and Veterinary Medicine, University of Miyazaki, Miyazaki 889-1692, Japan; piyarat_jiarpinitnun@med.miyazaki-u.ac.jp (P.J.); pornsawan_pongsawat@med.miyazaki-u.ac.jp (P.P.); kentaro-y@cc.miyazaki-u.ac.jp (K.Y.); 2 Miyazaki Prefecture Tsuno Meat Hygiene Inspection Center, Miyazaki 889-1201, Japan; 1959iwakiria5276@ezweb.ne.jp; 3 Laboratory of Veterinary Pathology, Department of Veterinary Medical Science, Faculty of Agriculture, University of Miyazaki, Miyazaki 889-2192, Japan; fuke.naoyuki.z2@cc.miyazaki-u.ac.jp; 4 Center for Animal Disease Control, University of Miyazaki, Miyazaki 889-2192, Japan; miyanishi.chizuru@cc.miyazaki-u.ac.jp (C.M.); sato1222@cc.miyazaki-u.ac.jp (S.S.); t_iwata@cc.miyazaki-u.ac.jp (T.T.); matsui.yuto.g4@cc.miyazaki-u.ac.jp (Y.M.); 5 Department of Veterinary Public Health, Chulalongkorn University, Bangkok 10330, Thailand; taradon.l@chula.ac.th

**Keywords:** *Campylobacter* spp., spotty liver disease (SLD), avian vibrio hepatitis (AVH), liver, broiler, poultry

## Abstract

Spotty liver disease (SLD) affects free-range laying hens, leading to mortality and reduced egg production. *Campylobacter* species, including *Campylobacter hepaticus*, have been associated with SLD cases worldwide. However, the cause of SLD-like lesions found in broilers in Japan still remains unclear. The present study aimed to investigate the involvement of *Campylobacter* spp. in broiler SLD by conducting microbiological, molecular biological, serological, histopathological, and immunohistopathological examinations using specimens of liver, bile, cecum, and serum from SLD-like and non-SLD chickens. *C. jejuni* was predominantly isolated and detected in approximately 40% of both non-SLD livers and SLD-like livers, with no significant difference between them. However, *C. hepaticus* was neither isolated nor detected in this study. Gross and histopathology revealed multifocal necrotizing hepatitis, suppurative granulomatous hepatitis, and cholangiohepatitis. Hepatitis stages are classified as no hepatitis, subclinical, acute, and chronic hepatitis. *C. jejuni* was more frequently present in acute-stage SLD-like livers, and high IgG antibody levels were noted in both subclinical and SLD-like-affected chickens, indicating *C. jejuni* infection. Immunohistochemical examination also supported these findings. The findings suggest that *C. hepaticus* was not involved in SLD-like in broilers in Japan, but *C. jejuni* translocation from the intestines to the liver may be a contributing factor.

## 1. Introduction

*Campylobacter* is a genus of Gram-negative, spiral-shaped bacteria that includes several species with zoonotic potential capable of causing animal illness [1]. Campylobacteriosis in humans is caused primarily by the consumption of contaminated food, particularly undercooked or contaminated poultry [2], unpasteurized milk [3], and untreated water [4]. *C. jejuni* is a common human pathogen that causes diarrheal illnesses [5]. Moreover, *Campylobacter* infection can lead to various complications such as hepatitis and cholecystitis [6], hemolytic uremic syndrome (HUS) [7], meningitis [8], fetal death [9], or Guillain-Barré syndrome (GBS) [10].

Chickens are primary reservoirs of *Campylobacter* spp., mainly *C. jejuni* and *C. coli*. Since chickens may harbor these organisms in the intestinal tract without any obvious clinical symptoms under appropriate husbandry conditions, they have a symbiotic relationship as part of the intestinal flora [11]. However, in the 1950s, avian vibrionic hepatitis (AVH) was reported worldwide in the poultry industry [12,13,14,15,16,17]. It affected laying hens and caused economic losses due to low egg production and high mortality [18,19]. AVH was characterized by multifocal degeneration and necrosis of the liver parenchyma [13,15,20]. After the 1970s, however, reports of AVH became fewer. *Vibrio*-like organisms, later classified as *Campylobacter*, were suggested as possible causative agents [21], but were not precisely identified.

In 2003, a disease very similar to AVH, characterized by white spotted lesions on the liver, was reported again in free-range laying hens in the UK [22] and named “spotty liver disease” (SLD) or “spotty liver disease syndrome” (SLS) in New Zealand, Australia, and other countries [22,23,24,25,26]. *Campylobacter*-like bacteria were isolated from the liver, bile, and intestinal contents, and the lesion was successfully reproduced through experimental infection [27]. These isolates were later named as a new *Campylobacter* species, *C. hepaticus* [28].

According to the livestock statistics of the Ministry of Agriculture, Forestry and Fisheries (MAFF), Japan, as of 1 February 2023 there were approximately 128 million adult layer chickens and 141 million broiler chickens housed in Japan [29]. There have been no reports of SLD in laying hens in Japan, but SLD-like lesions were found in broilers during poultry meat inspections in the 1990s. This increased detection rate followed the enactment of the Poultry Inspection Law in 1990 and the mandate of the Ministry of Health, Labor and Welfare (MHLW) for individual inspections of chickens, ducks, and turkeys at large poultry processing plants [30]; however, most of the birds inspected in Japan were broilers.

In Japan, AVH-like lesions are recorded as “inflammation” during poultry meat inspection. Affected broilers exhibit white spots (focal necrosis) on the liver, and this may vary from farm to farm. In some cases, many AVH-like lesions are observed simultaneously on the same farm. Bacteriological tests targeting *Campylobacter* species have been conducted. Still, it has remained inconclusive whether *C. jejuni* was the major causative agent of the AVH because it was isolated from both AVH-affected chickens and grossly normal livers and bile (unpublished data). Since 2000, such outbreaks have declined, due to countermeasures for preventing high pathogenic avian influenza such as improved feeding environment, enhanced biosecurity, and reduced chicken housing density. Although SLD-like lesions continue to be found in broilers during meat inspection, albeit at low rates, the involvement of *C. hepaticus* in liver lesions has not been investigated in Japan, and the causative agent remains unknown.

The present study conducted bacteriological tests for *Campylobacter* species on broilers affected by SLD-like and with grossly normal livers. In addition, molecular biological, pathological, histopathological, immunohistochemical, and serological tests were performed to investigate whether *Campylobacter* species, including *C. hepaticus*, are involved in SLD in broilers.

## 2. Materials and Methods

### 2.1. Sample Collection

Following the standard procedures for slaughtering and evisceration as outlined in the Poultry Slaughtering Business Control and Poultry Inspection Act under the Ministry of Health, Labour and Welfare of Japan, samples of the liver with the gallbladder, cecum, and blood clots in the heart were collected by meat inspectors (veterinarians) from broiler chickens around seven weeks of age without any clinical disorders at a chicken processing plant located in Miyazaki Prefecture, Japan, between September 2021 and April 2023. A total of 53 livers with no gross lesions (referred to as non-SLD) and 66 livers with SLD-like lesions (referred to as SLD) were randomly collected, and bile samples were taken aseptically from the gallbladders. Cecum samples from non-SLD (n = 33) and SLD (n = 35) were also examined. These samples were subjected to bacteriological, molecular biological, pathological, and immunohistochemical tests. To detect antibodies against *Campylobacter* spp., 20 non-SLD and 20 SLD (n = 20) chickens were selected based on the results of bacteriological and multiplex PCR tests of all the livers collected, and serum samples were collected from blood clots in the heart. All samples were placed in sterile bags and promptly transferred to the laboratory in insulated boxes with ice packs, and testing was initiated within 24 h of collection.

### 2.2. Isolation of Campylobacter Species

*Campylobacter* spp. was isolated using a combination of direct plating and enrichment culture followed by plating on *Campylobacter*-selective agar plates. The surface of the liver capsule was decontaminated with 70% (*v*/*v*) ethanol, and then the surface was subjected to a brief thermal treatment with a burner, aseptically cut, and the deep liver parenchymal split surface was stamped onto two selective media for *Campylobacter* spp., which included Brucella agar (BBL Microbiology Systems, Cockeysville, MD, USA) with 5% (*v*/*v*) horse blood supplemented with Skirrow *Campylobacter* Selective Supplement (Thermo Scientific Oxoid, Hampshire, UK) and modified charcoal cefoperazone deoxycholate agar (mCCDA) (Thermo Scientific Oxoid). These plates were incubated at 37 °C under microaerophilic conditions (80% N_2_, 10% CO_2_, 5% O_2_, and 5% H_2_) for up to seven days. Additionally, an aliquot of 20 μL of bile and 0.2 g of cecum content were cultured on the same selective *Campylobacter* media described above. Growth of suspected *Campylobacter* spp. on the selective agar plates was observed at 3-, 5-, and 7-days post-cultivation.

For enrichment culture, approximately 1 g of liver, 1 g of swabbed cecum content with a cotton swab, or 100 μL of bile was inoculated in 9 mL of Preston enrichment broth (Thermo Scientific Oxoid) supplemented with 5% (*v*/*v*) lysed horse blood and then incubated at 37 °C for 48 h under microaerobic conditions. Subsequently, a loopful (approximately 10 μL) of the enrichment culture was streaked onto mCCDA agar plates, and incubated at 37 °C for 48 h. When these culture methods isolated *Campylobacter*, the specimen was recorded as positive for isolation. Three suspected *Campylobacter* spp. colonies were identified after Gram staining and biochemical tests, and ambiguous isolates were subjected to PCR for species-specific confirmation.

To enumerate campylobacters in the cecum contents, direct plating was used. Ten-fold serial dilutions of each specimen (0.2 g) were made in 10 mM phosphate-buffered saline (pH 7.2, PBS), 25 μL aliquots of each dilution were plated onto mCCDA agar, and incubation was carried out at 37 °C for 48 h. Colonies were counted to determine colony-forming units (cfu/g), with a lower detection limit of 4 × 10^2^ cfu/g. Up to three suspected colonies from plates with the highest dilutions were subcultured onto new mCCDA plates and confirmed in the same way as for direct plating and enrichment culture. Suspected *Campylobacter* spp. isolates were preserved in Brucella broth (BBL Microbiology Systems) containing 10% (*v*/*v*) glycerol (Nakarai Tesque Inc., Kyoto, Japan) at −80 °C for further analysis. *C. jejuni* 81-176 (NCTC 11828), *C. coli* JCM2529 (NCTC 11366), and *C. hepaticus* NCTC 13,823 were used as positive controls.

### 2.3. Multiplex PCR for Detection of Campylobacter Species

Bacterial DNA was extracted from pure cultured colonies isolated from liver, bile, and cecum contents using an alkaline-heat extraction method [31]. Moreover, DNA was extracted from Preston enrichment broth after cultivation by heating the precipitate from the broth [32]. DNA from the liver tissue was extracted using DNeasy blood and tissue Kits (Qiagen Ltd., Hilden, Germany) following the manufacturer’s instructions. For extraction of DNA from the bile and cecum contents, the QIAamp DNA Stool Mini Kit (Qiagen) was used for the removal of PCR inhibitors. The extracted DNA samples were collected and stored at −20 °C until testing.

To differentiate the various *Campylobacter* species, multiplex PCR was performed using a combination of previously reported primers [33,34,35,36], including those for the genus *Campylobacter* (C412F and C1228R for the 16S rRNA gene, 816 bp), *C. jejuni* (C-1 and C-3 for the cj0414 gene, 161 bp), *C. coli* (CC18F and CC519 for the *ask* gene, 502 bp), and *C. hepaticus* (G2F3 and G2R2 for the *glk* gene, 463 bp). All primers were produced by Eurofins Genomics Co., Ltd. (Tokyo, Japan). Positive controls included *C. jejuni* 81-176, *C. coli* JCM2529, and *C. hepaticus* NCTC 13823.

PCR was performed in a DNA thermal cycler (SimlpiAmpTM Thermal Cycler, Thermo Fisher Oxoid) in 20 μL of the reaction mixture. Each mixture contained 2.0 μL (approximately 20 ng) of genomic DNA, 2.0 μL of 10 × reaction buffer (Qiagen), four primer sets at 160 nM each, 0.625 U of Taq DNA polymerase (Qiagen), each of the deoxynucleotides at 250 μM (Amersham Pharmacia Biotech, Tokyo, Japan), and DNase-free distilled water (DW). The samples were incubated at 95 °C for 1 min to denature the target DNA, followed by 35 cycles of 95 °C for 1 min, annealing at 56 °C for 1.5 min, and 72 °C for 1 min, with a final incubation at 72 °C for 7 min. Amplicons were held at 4 °C before analysis. Each reaction mixture was analyzed by gel electrophoresis through 1.5% (*w*/*v*) agarose (Nakarai Tesque) in 1 × Tris-acetate buffer (TAE) (Cosmo Bio Co., Ltd., Tokyo, Japan) and visualized by UV transillumination using Chemidoc™ Touch MP (Bio-Rad Laboratories Inc., Hercules, CA, USA) after staining with gel red (Biotium Inc., Fremont, CA, USA).

### 2.4. Pathological Examination

**Figure 1:**
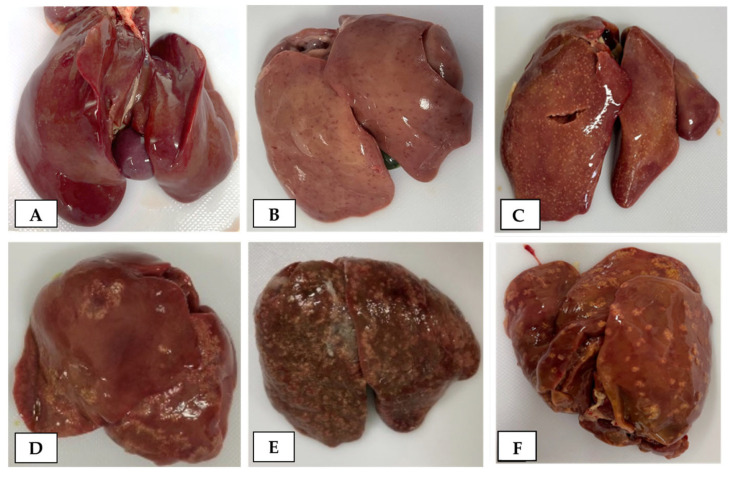
Gross pathological features of livers collected from non-SLD and SLD chickens. (**A**) normal surface appearance in non-SLD chickens; (**B**,**C**) multiple 1–2-mm-diameter spots distributed in a few areas or the whole liver surface, indicating mild-grade SLD; (**D**) multiple spots larger than 2 mm in diameter distributed in a few areas of the liver surface, indicating moderate-grade SLD; and (**E**,**F**) multiple spots larger than 2 mm in diameter distributed over the whole liver surface, indicating severe-grade SLD.

**Figure 2:**
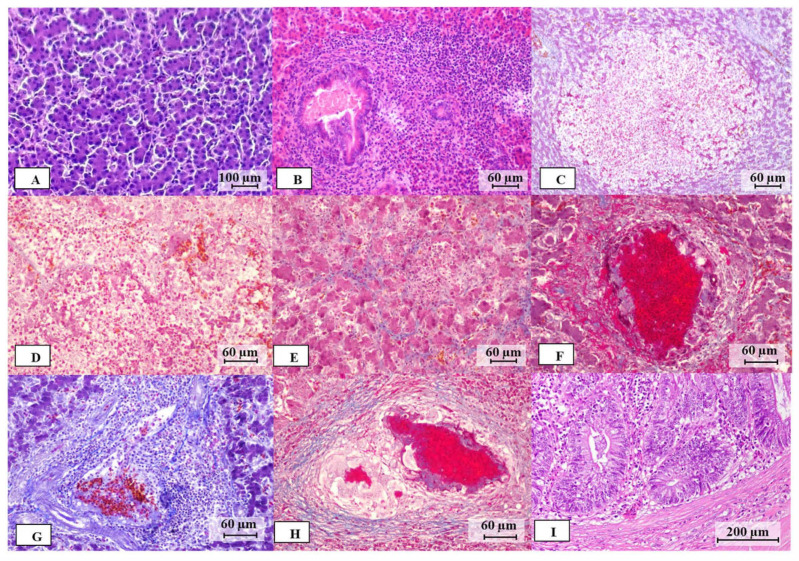
Histological appearance of liver and cecum samples collected from non-SLD and SLD chickens: (**A**) HE staining of a normal liver; (**B**) HE staining showing mild hepatocellular degeneration with periportal inflammation; (**C**,**D**) Masson’s trichrome staining of livers with acute hepatitis; (**E**–**H**) Masson’s trichome staining of livers with chronic hepatitis. Additionally, (**I**) shows HE staining of the cecum with inflammatory cell infiltration in the submucosa, lamina propria, and crypts of villi, with enterocyte necrosis.

**Figure 3:**
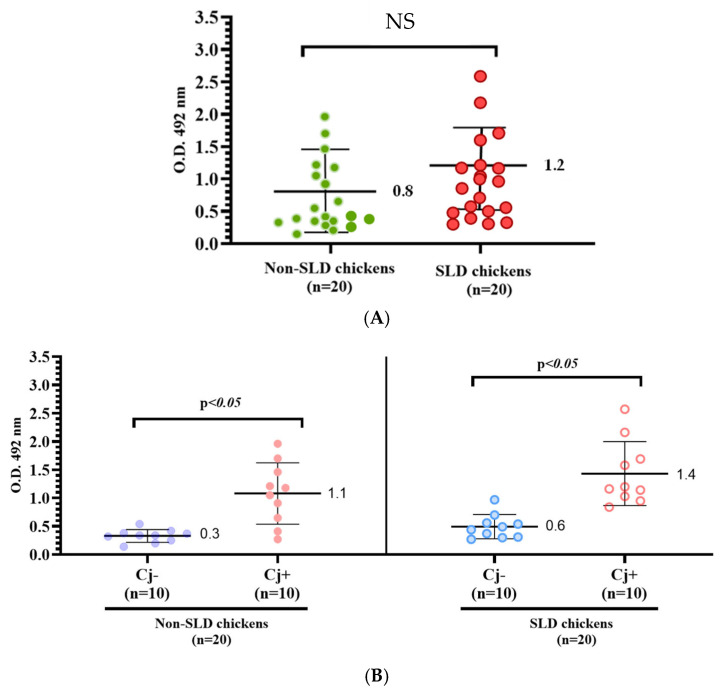
Comparative analysis of IgG antibody levels to *C. jejuni* in non-SLD and SLD broilers (**A**) and in those with and without *C. jejuni* isolation and/or detection in the liver (**B**). NS: Not significant.

Before the microbiological examination, the livers were subjected to gross pathological examination to classify them based on surface features (Figure 1) as follows: (1) non-SLD (no lesions evident, Figure 1A); (2) mild-grade SLD (a few spots 1–2 mm in diameter distributed anywhere on the liver surface, Figure 1B,C); (3) moderate-grade SLD (spots larger than 2 mm distributed over several areas of the liver surface, Figure 1D); and (4) severe-grade SLD (larger spots distributed over the entire liver surface, resembling cauliflower-like formations, Figure 1E,F). Gallbladder and cecum samples were also examined for any lesions. After the gross pathological examination, a histopathological examination was performed. Liver and cecum samples were fixed in a 10% neutral buffered formalin solution (Nakarai Tesque) and embedded in paraffin (Sakura Finetek Japan Co., Ltd., Tokyo, Japan). Thin sections 4–5 μm thick were then prepared and stained with hematoxylin-eosin (HE) for general histology. Masson’s trichome staining was conducted to detect fibrin or fibrosis within liver tissues. Histopathological observations were made using a microscope to evaluate hepatitis stages in both SLD and non-SLD liver tissue. Hepatitis stages were classified as follows based on previously reported criteria: (1) no hepatitis, (2) subclinical hepatitis, (3) acute hepatitis, and (4) chronic hepatitis [37]. The histopathological changes included hepatocellular degeneration or necrosis, erythrocyte infiltration, fibrin deposition, and the types of inflammatory cells (lymphocytes, heterophils or macrophages) infiltrating the liver parenchyma and periportal areas. Subclinical hepatitis was identified by the presence of hepatocyte degeneration and cellular infiltration in peripheral areas. Acute hepatitis was characterized by fibrin deposition, and cellular infiltrates were observed in both peripheral areas and within the liver parenchyma. Chronic hepatitis was characterized as granulomatous necrotizing hepatitis with peripheral infiltration. Granulomas were examined for centers of caseous debris or fibrin, surrounded by multinucleated giant cells (macrophages) and other inflammatory cells, and areas of fibrous tissue deposition [37].

### 2.5. ELISA Detection of Serum IgG Antibody Against C. jejuni

Serum IgG antibodies against *C. jejuni* were measured by ELISA to predict *C. jejuni* infection in chickens with and without SLD. *C. jejuni* 81-176 (Penner serotype 23, 36) [38,39] was used as the antigen for the ELISA, and 20 samples from each of the SLD and non-SLD broilers were collected. Among the 20 samples, 10 were *C. jejuni* positive (Cj+) and 10 were negative (Cj−) based on the culture and/or PCR results. The heart of each chicken was aseptically opened, and blood clots were collected in a 1.5-mL microtube. The blood samples were incubated at 37 °C for 1 h, and then the serum was separated by centrifugation at 8000× *g* for 15 min at 4 °C. The serum samples were stored at −20 °C until tested. As described previously, soluble antigens were obtained by acid extraction and ELISA [40]. For measurement of the IgG antibodies against *C. jejuni*, each well of a 96-well ELISA plate (Thermo Fisher Scientific, Waltham, MA, USA) was coated with 75 μL of soluble antigen (1.0 μg/mL) in 10 mM PBS and incubated at 37 °C for 1 h. After discarding the antigen, non-binding sites were blocked with 200 μL of 1% (*w*/*v*) bovine serum albumin (Sigma-Aldrich Japan Co., LLC., Tokyo, Japan) in PBS, incubated at 37 °C for 1 h, and then again at 4 °C for 18 h. After washing with PBS containing 0.05% (*v*/*v*) Tween 20 (PBST) (Nakarai Tesque), 75 μL of 100-fold-diluted chicken serum with a blocking solution was added. PBS was used as a control. Plates were incubated at 37 °C for 1 h, then washed with PBST, incubated with rabbit anti-chicken IgG γ chain antibody conjugated with horseradish peroxidase (Thermo Fisher Scientific) diluted 1000-fold in blocking solution, and incubated at 37 °C for 1 h. After washing, 75 μL of a chromogenic substrate containing 3 mM O-phenylenediamine (Nakarai Tesque) in 20 mM citric acid buffer (pH 5.0) was added and incubated at 20 °C for 30 min. The reaction was stopped by adding 75 μL of 1 M sulfuric acid (Nakarai Tesque). The optical density was measured at 492 nm (OD492) using a Bio-rad Benchmark (Bio-Rad Laboratories, Hercules, CA, USA).

### 2.6. Immunohistopathological Examination

Immunohistochemistry (IHC) was conducted to detect *C. jejuni* antigens in liver tissues [41]. Ten liver specimens from each of the SLD and non-SLD chickens were selected from Cj+ and Cj− specimens. Thin sections of the liver and cecum prepared for histopathological examination were placed on platinum-coated slide glasses (Matsunami Glass Ind., Ltd., Osaka, Japan). Sections were deparaffinized with 100% (*v*/*v*) xylene three times for 10 min each, rehydrated with 80–100% (*v*/*v*) ethanol for 30 s each, and antigen retrieval was performed by autoclaving at 105 °C for 10 min. After cooling under running tap water for 20 min, the sections were washed thrice with PBS for 5 min each. Endogenous peroxidase activity was inhibited by treating the sections with methanol containing 3% (*v*/*v*) H_2_O_2_ for 30 min at 20 °C followed by washing in PBS. To prevent non-specific binding, the sections were treated with Blocking One solution (Nakarai Tesque) at 20 °C for 30 min, then washed with PBS. As primary antibodies, polyclonal rabbit anti-*C. jejuni* antisera (*C. jejuni* strains 81-176 and OH4382) diluted 1:2000 were applied to the sections, and incubation was performed at 37 °C for 1 h, followed by washing in PBS. As a negative control, only PBS was applied to the sections. After PBS washing, goat anti-rabbit IgG labeled with horseradish peroxidase (Envision polymer; Dako Inc., Carpinteria, CA, USA) was applied and incubated at 37 °C for 40 min. The sections were washed with PBS and stained with 3,3′-diaminobenzidine (Dako) to visualize the antigen–antibody complexes. The reaction was stopped by washing the slides with DW at 20 °C for 5 min. The slides were then counterstained with hematoxylin for 20 s, dehydrated with 70–100% (*v*/*v*) ethanol for 30 s each, and treated with 100% (*v*/*v*) xylene three times for 15 min each. Bacterial antigens were observed using a light microscope. The positive control was used for cecum tissue from which *C. jejuni* had been isolated. Antigen detection areas were observed and counted per section, then compared between SLD and non-SLD in both the Cj+ and Cj− groups with high and low IgG antibodies.

### 2.7. Statistical Analysis

All statistical calculations were performed using GraphPad Prism 10 software version 10.2.2 for Windows (GraphPad Software Inc., Boston, MA, USA). Bacterial counts were converted into base-10 logarithms of cfu per gram (log10 cfu/g). The rates of *Campylobacter* spp. isolation and detection were expressed as percentages, with samples categorized as Cj+ (where *C. jejuni* was isolated or detected) and Cj− (where *C. jejuni* was not found). IgG ELISA titers were expressed as averages with standard deviation. Differences in IgG ELISA titers were analyzed using one-way ANOVA and Tukey’s multiple comparisons test. The proportions of bacterial isolation rates by cultivation, detection rates by PCR, and areas of antigen detection per section were compared using contingency tables, the chi-squared test, and Fisher’s exact test. Statistical significance was defined as *p* < 0.05.

## 3. Results

### 3.1. Isolation of Campylobacter spp.

The rates of isolation by cultivation and the rates of detection by multiplex PCR for *Campylobacter* spp. are summarized in Table 1. *C. jejuni* was the predominant species isolated and detected in both non-SLD and SLD chickens, regardless of gross liver lesions. In non-SLD chickens, the rates of *C. jejuni* isolation from the liver, bile, and cecum contents were 43.4%, 18.9%, and 36.4%, respectively, while in SLD chickens, the corresponding rates were 40.9%, 15.2%, and 22.9%, respectively (Table 1). *C. coli* was isolated with lower frequency from the liver, bile, and cecum contents. *C. jejuni* was isolated from all livers from which *C. coli* had been isolated. Multiplex PCR showed slightly higher detection rates for both *C. jejuni* and *C. coli* than for isolation across all samples. There were no statistically significant differences between non-SLD and SLD chickens with regard to the isolation and PCR detection rates for *C. jejuni* from the liver, bile, and cecum contents. *C. hepaticus* was not isolated from any samples by either cultivation or PCR. Quantitative analysis of *Campylobacter* in the cecum contents revealed no significant difference between non-SLD and SLD chickens, with levels ranging from 7.2 to 8.0 log cfu/g (median: 7.6 log cfu/g) and from 7.1 to 8.1 log cfu/g (median: 7.7 log cfu/g), respectively.

### 3.2. Pathological Observations

SLD chickens revealed spots throughout the liver surface, varying according to area and degree, as shown in Figure 1. The stages of non-SLD and SLD were classified based on gross and histopathological features of the liver (Table 2). Based on gross pathological findings, livers from SLD chickens were classified as mild-grade (25.8%), moderate-grade (42.4%), and severe-grade SLD (31.8%), respectively. Moreover, gallbladder enlargement was observed in 15.2% of SLD chickens. Swelling of the cecum was observed in both SLD (22.9%) and non-SLD (36.4%) chickens.

Histopathological features of non-SLD and SLD livers included hepatocyte degeneration, erythrocyte infiltration, and inflammatory cell infiltration, as shown in Figure 2. In livers with no gross lesions, histopathological examination revealed no specific features in 19 of 53 livers (35.8%), while 34 livers (64.2%) showed hepatocyte degeneration, erythrocyte infiltration, and periportal inflammation referred to as cholangiohepatitis, suggesting a subclinical stage (Table 2). SLD livers showed cholangiohepatitis and multifocal necrotizing hepatitis (MNH) with or without fibrosis (Table 2). SLD livers with acute hepatitis (25.8%) showed cell infiltration (mainly erythrocytes, heterophils, and lymphocytes) and fibrin deposition without fibrosis. Chronic-stage SLD livers (74.2%) exhibited varying degrees of fibrosis alongside inflammatory cell infiltration (lymphocytes, heterophils, and mainly macrophages) (Table 2). Bile duct proliferation and bile accumulation were observed in both SLD (56.1%) and non-SLD (54.7%) chickens. In the swollen cecum, an inflamed mucosal area with crypt hyperplasia and villous atrophy was widespread in all of the specimens examined (Figure 2).

The rates of *C. jejuni* isolation and detection in non-SLD and SLD livers classified based on gross and histopathological findings were collated. *C. jejuni* was not isolated or detected in the livers of non-SLD chickens with no apparent abnormalities. On the other hand, the isolation/detection rate of *C. jejuni* from livers with cholangiohepatitis was 43.4%, which was the highest among the samples including livers from SLD chickens. Among the livers from SLD chickens with acute and chronic stages of hepatitis, the isolation/detection rate of bacteria was roughly 11–17%, with no significant difference between the various stages of hepatitis (Table 2).

### 3.3. Serum IgG Antibody Against C. jejuni

An ELISA assay was performed to deduce whether broilers had been infected with *C. jejuni* regardless of the presence or absence of SLD lesions in the liver. When the IgG levels against *C. jejuni* between non-SLD and SLD broilers were compared, there was no significant difference between the two groups (Figure 3A). However, when IgG antibody levels were compared between chickens in which *C. jejuni* was isolated and/or detected from livers (Cj+) and those in which it was not (Cj−), significant differences were found in both non-SLD and SLD chickens (Figure 3B). In both groups, IgG antibody titers were significantly higher in the group of Cj+ than in that of Cj−.

### 3.4. Immunohistochemical Examination

**Figure 4:**
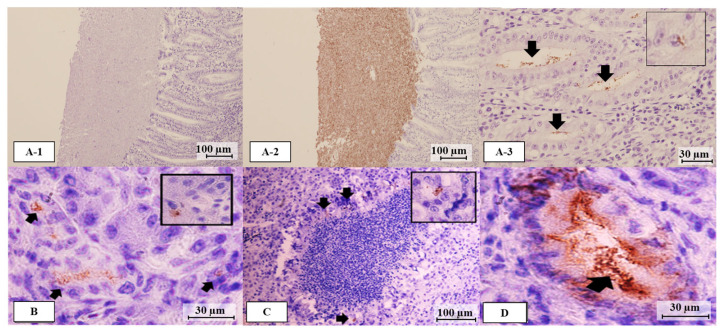
Immunohistochemical detection of *C. jejuni* antigen from non-SLD and SLD chickens. (**A-1**) negative control; (**A-2**) positive control with *C. jejuni* antigens detected in cecum content; (**A-3**) *C. jejuni* antigens in crypts of villi (black arrows) and epithelial cells (small square); (**B**) *C. jejuni* antigens in macrophages (black arrows) and hepatocytes (small square) of non-SLD chickens; (**C**) *C. jejuni* antigens in hepatocytes surrounding necrotizing lesions (black arrows) and macrophages (small square) of SLD chickens; (**D**) *C. jejuni* antigens in bile duct from a bile-positive sample.

The detection of *C. jejuni* antigen by immunohistochemistry from non-SLD and SLD chickens is shown in Figure 4. Although antibodies of two Penner serotypes (OH4382 and 81-176 strains) were used to detect the antigens, no difference in antigen distribution was observed. Cecum tissue from which *C. jejuni* was isolated was used as a positive control to verify the immunohistochemical test conditions, confirming abundant antigen detection in epithelial crypts (Figure 4(A-2,A-3)). SLD and non-SLD livers were observed within five views per section. The antigen distribution area of individual samples is shown in Table 3.

Non-SLD chickens were defined as those without gross lesions in the liver but included those in which *C. jejuni* was not isolated or detected in the liver and no histopathological lesions were observed (normal) and those in which *C. jejuni* was isolated or detected in the liver and cholangiohepatitis was evident (subclinical stage). No *C. jejuni* antigen was detected in the liver tissue of the former. In contrast, in the latter, *C. jejuni* antigen was detected in hepatocytes located around necrotic tissue, inflammatory cells, and capillary bile ducts but not within the necrotic tissue itself (Figure 4B–D). However, the distribution of the antigen was low. In addition, the IgG antibody levels of chickens in which *C. jejuni* antigen was detected were higher than those of chickens in which the antigen was not detected (Table 3). SLD chickens had liver tissue showing both the acute and chronic stages. In those at the acute stage, *C. jejuni* was isolated and detected in all five SLD liver samples, while in those at the chronic stage, *C. jejuni* was detected by PCR in two of the five specimens tested. Examination of the antigen distribution of *C. jejuni* in liver tissues at the acute and chronic stages revealed that it was present in all of the acute-stage tissues examined, as was the case for the subclinical stage, although no antigen was detected in liver tissues at the chronic stage. IgG antibody levels were higher in chickens in which *C. jejuni* had been isolated and/or detected in the liver (Table 3).

## 4. Discussion

The objective of this study was to determine whether *Campylobacter* spp. including *C. jejuni, C. coli,* and *C. hepaticus* are involved in the incidence of SLD in Japan as reported in other countries. However, *C. hepaticus* was not isolated from the liver, bile, or cecum contents of broilers without gross lesions or those showing SLD, nor was it detected by PCR. These findings suggest that *C. hepaticus* may not be involved in SLD lesions in broilers, at least in Miyazaki Prefecture, Japan. In contrast, *C. jejuni* and *C. coli* were isolated and detected at similar rates in both SLD and non-SLD broilers, making it challenging to determine their role in SLD. Several reports have indicated the possibility of internal and external liver contamination by *Campylobacter* spp. [42,43]. However, it is unlikely that *Campylobacter* would have been isolated and detected on the liver surface because the fresh liver samples used in this study were subjected to surface decontamination with 70% (*v*/*v*) ethanol and burning surface of the liver for a few seconds, followed by aseptic cutting.

Several key questions arise regarding the involvement of *C. jejuni*/*coli* in the formation of white spots (focal necrosis) on broiler chicken livers. These include the routes by which *C. jejuni*/*coli* reaches the liver and gallbladder, whether *C. jejuni*/*coli* has the pathogenic potential to cause the observed focal necrosis, reasons for the absence of *C. jejuni*/*coli* in all of the SLD-affected livers, and whether the isolation and detection of *C. jejuni*/*coli* in grossly normal livers represents an early or subclinical infection stage. To address these questions, to ascertain the possibility of prior infection with *C. jejuni*, an IHC test was employed to determine the distribution of *C. jejuni* antigens in liver tissue, and the IgG antibody levels in serum against *C. jejuni* were measured. Since the pathogenicity of *C. jejuni* has been studied in greater detail than that of *C. coli*, and most of the isolates included in this bacteriological examination were *C. jejuni*, the discussion will be focused on this species.

Previous studies have offered intriguing insights when considering the route of *C. jejuni* to the liver. Experimental studies have shown that *Campylobacter* spp. can be recovered from the livers of specific pathogen-free chickens after oral inoculation [44,45], resulting in bacterial invasion into the intestinal mucosa followed by translocation to extraintestinal tissues such as the liver and spleen [44,45,46]. These migration routes likely occur through the biliary, lymphatic, or vascular systems [47]. The potential for *C. jejuni* to induce hepatitis by translocating from the intestinal tract to the hepatobiliary system, leading to bacteremia and focal necrosis in the liver, is further supported by experimental infection in various animal models (rhesus monkeys, mice, and quails) and a case report in humans [48,49,50,51]. Notably, in a study reported by Misawa et al. [50], *C. jejuni* strains isolated from the liver with SLD-like lesions in a broiler chicken and a human patient with diarrhea were used to infect quail. The *C. jejuni* was inoculated intravenously and successfully reproduced SLD-like lesions in the liver, indicating that *C. jejuni* has the potential to cause focal liver necrosis. At large poultry farms, broilers are usually housed under high-density conditions, making them susceptible to stress due to poor ventilation, high temperature, and other factors. Stress is known to promote translocation of intestinal bacteria [52], and such a rearing environment may also be an important factor related to extraintestinal infection.

In contrast to our initial expectations, *C. jejuni* was isolated and detected from non-SLD livers as well as SLD livers in the 40% range, with no significant difference between the two. The isolation of *C. jejuni* from non-SLD livers can be explained by the findings of histopathological and immunohistological examinations, and the levels of IgG antibody against *C. jejuni*. Observation of histological sections of the liver without grossly visible lesions revealed no significant histopathology in 19 of 53 livers (35.8%), while 34 of the livers (64.2%) showed hepatocyte degeneration, erythrocyte infiltration, and periportal inflammation (Table 2). In the former case, *C. jejuni* was not isolated or detected in the liver, no histopathological abnormality was observed, and no bacterial antigen was detected. Furthermore, the absorbance at OD429, which indicates the level of IgG antibody against *C. jejuni*, was less than 0.4, suggesting that the liver was normal and might not have been infected with *C. jejuni*. Conversely, in the latter case, *C. jejuni* was isolated and detected, bacterial antigens were identified, and the levels of IgG antibody against *C. jejuni* were higher than 0.9 at OD429. Furthermore, pathological examination revealed the presence of an inflammatory cell infiltrate. Thus, even without gross lesions in the liver, the histological evidence of inflammation, the presence of *C. jejuni* antigens in the liver tissue, and the increased level of serum IgG antibodies suggested that these chickens were infected with *C. jejuni* and had subclinical SLD. These results show that even if the liver appears normal and has no obvious lesions, it could harbor *Campylobacter*, putting public health and food safety at risk.

In discussing the reasons why *C. jejuni* was not isolated and detected in all of the livers showing SLD and in non-SLD livers at the subclinical stage, it is necessary to consider the viability and proliferative potential of *C. jejuni* in the extraintestinal tract. Kiehlbauch et al. examined phagocytosis and intracellular survival of *C. jejuni* strains in human and mouse mononuclear phagocytes in vitro and found that the bacteria survived intracellularly for up to 6–7 days [53]. De Mero et al. [54] also examined the intracellular survival of *C. jejuni* during infection of HEp-2 cells in vitro. This revealed that *C. jejuni* strains were attached to the HEp-2 cell membrane, internalized by a phagocytosis-like mechanism, but digested after phagosome-lysosome fusion after 36 h. In the present study, the absence of antigens within the centers of necrotic areas suggested that *C. jejuni* did not proliferate in the liver [55]. These findings suggest that *C. jejuni* can survive intracellularly for only a short period, eventually being killed by the host’s response, resulting in rapid clearance or translocation to bile ducts but triggering an immune response leading to inflammatory cell infiltration, fibrosis, and focal necrosis.

In addition to cell adherence and invasiveness, other virulence factors of *C. jejuni* have been reported to include toxin production that exhibits hepatotoxic activity [55]. *C. jejuni* cells that are translocated from the intestinal tract to the liver have the potential to invade Kupffer cells and hepatocytes. Although they are unable to survive intracellularly for extended periods, these bacteria are postulated to be capable of causing cell necrosis through cytolethal activity of cells in the liver. The relatively high rate of *C. jejuni* isolation from the liver in the absence of gross lesions may be attributed to repeated translocation from the intestinal tract before raising host immunity. This may result in hepatocyte damage even in the absence of bacterial multiplication within the liver tissue. Additionally, host responses may influence the pathological magnification. Further studies in vitro and in vivo are required to elucidate the pathogenic significance of *C. jejuni* in non-SLD livers.

Histopathological examination of liver specimens showing SLD lesions allowed classification into acute and chronic stages, depending on the infiltrating cells and the degree of fibrosis (Figure 2 and Table 2). Interestingly, *C. jejuni* was isolated and detected by PCR from all SLD livers at the acute stage, but from only two samples at the chronic stage. Judging from the high levels of IgG antibodies against *C. jejuni* in chickens at the chronic stage as well as at the acute stage, it was considered that the chickens were infected with *C. jejuni*. Histopathological examination of the liver in the chronic phase demonstrated significant deposition of fibrous tissue as a healing response after long-term inflammation and foreign body encapsulation, suggesting that host defense reactions, including the immune response, may have reduced the presence of *C. jejuni* to below the detectable level.

Considering the etiology of necrotizing hepatitis lesions in broilers, other pathogens may also contribute to SLD. Previous research has identified *C. billis* [56], and a galliform *Chaphamaparvovirus* [57] as potential causative agents. Other bacteria, including *Clostridium perfringens*, *Escherichia coli*, *Salmonella*, and *Pasteurella*, can also induce hepatitis and liver surface abscesses in poultry [58]. However, the proliferation profile in the liver may differ among bacterial species. In chicks, the immune system is not fully developed at hatching, taking several weeks to mature [59]. During this time, broiler chicks may be exposed to many organisms before they reach slaughtering age. Accordingly, the present study implies that the reasons for the absence of *C. jejuni* antigen in liver tissue and the observation of low antibody titers in chickens in the chronic stage of hepatitis may have included the influence of other microorganisms or other causes.

## 5. Conclusions

Our findings suggest that *C. hepaticus* may not be associated with SLD in broiler chickens, at least in Miyazaki, Japan. As to whether *C. jejuni* is involved in SLD in broilers, not only bacteriological but also histopathological, immunohistological, and serological tests are necessary. The present results show that *C. jejuni* translocated from the intestinal tract to the extraintestinal tract, including the liver tissue. Furthermore, SLD was shown to have subclinical, acute, and chronic stages, and *C. jejuni* and its antigen were isolated or detected in broilers at the subclinical and acute stages, which showed high levels of antibody against the organism. These findings suggest that *C. jejuni* is invasive to liver tissue and has the potential to cause focal necrosis as a result of various host defense responses.

## Figures and Tables

**Table 1:** Rates of *Campylobacter* spp. isolation and detection by cultivation and multiplex PCR from non-SLD and SLD chickens.

Species	Liver				Bile				Cecum Content			
	Non-SLD (n = 53)		SLD (n = 66)		Non-SLD (n = 53)		SLD (n = 66)		Non-SLD (n = 33)		SLD (n = 35)	
	Isolation *	PCR **	Isolation *	PCR **	Isolation *	PCR **	Isolation *	PCR **	Isolation *	PCR **	Isolation *	PCR **
*C. jejuni* (%)	23 (43.4)	23 (43.4)	27 (40.9)	30 (45.5)	10 (18.9)	17 (32.1)	10 (15.2)	12 (18.2)	12 (36.4)	15 (45.5)	8 (22.9)	10 (28.6)
*C. coli* (%)	1 (1.9)	6 (11.3)	1 (1.5)	2 (3.0)	0 (0.0)	0 (0.0)	0 (0.0)	0 (0.0)	2 (6.1)	4 (12.1)	0 (0.0)	1 (2.9)
*C. hepaticus* (%)	0 (0.0)	0 (0.0)	0 (0.0)	0 (0.0)	0 (0.0)	0 (0.0)	0 (0.0)	0 (0.0)	0 (0.0)	0 (0.0)	0 (0.0)	0 (0.0)

No significant difference in isolation (*) and detection (**) rates between non-SLD and SLD chickens.

**Table 2:** Pathological classification of non-SLD and SLD livers based on gross and histopathological findings and results of isolation and detection of *C. jejuni* from the liver at each stage.

Chicken	Gross Pathological Findings	Hepatitis Stage	Histopathological Findings	No. of Liver (%)	*C. jejuni*	
					Isolation (%)	PCR (%)
Non-SLD (n = 53)	None	No hepatitis	None	19 (35.8)	0 (0.0) *	0 (0.0) *
	None	Subclinical	Ery, Lym, Het, Mac	34 (64.2)	23 (43.4) **	23 (43.4) **
SLD (n = 66)	Mild-grade SLD	Acute	MNH with Ery, Lym, Het, Mac, FB, FT(±)	17 (25.8)	10 (15.2) ***	10 (15.2) ***
	Moderate-grade SLD	Chronic	MNH with Ery, Lym, Het, Mac, FB, FT(+)	28 (42.4)	10 (15.2) ***	11 (16.7) ***
	Severe-grade SLD		MNH with Ery, Lym, Het, Mac, FB, FT(++)	21 (31.8)	7 (10.6) ***	9 (13.6) ***

*, **, *** significant differences between histopathological classifications at *p* < 0.05. Ery: erythrocytes, FB: fibrosis, FT: fibroblast, Het: heterophils, Lym: lymphocytes, Mac: macrophages, MNH: Multifocal necrotizing hepatitis. ++: severe fibrosis; +: moderate fibrosis; ±: without or mild fibrosis.

**Table 3:** ELISA IgG titer and immunohistochemical results classified by hepatitis stages and bacteriological rates from non-SLD and SLD livers.

Liver	Hepatitis Stage	Sample No.	*C. jejuni*		ELISA(OD_429_)	Antigen Distribution Area				
			Isolation	PCR		Necrotic Area	Normal Area	Hepatocyte	Macrophage	Bile Duct
Non- SLD (n = 10)	No hepatitis (n = 5)	128L	−	−	0.1	−	−	−	−	−
		129L	−	−	0.2	−	−	−	−	−
		125L	−	−	0.3	−	−	−	−	−
		116L	−	−	0.3	−	−	−	−	−
		126L	−	−	0.4	−	−	−	−	−
	Subclinical hepatitis (n = 5)	113L	+	+	0.9	−	+	+	−	+
		118L	+	+	1.2	−	+	+	+	−
		132L	+	+	1.2	−	±	±	+	−
		97L	+	+	1.7	−	+	+	+	−
		108L	+	+	2.0	−	+	±	+	−
SLD (n = 10)	Acute hepatitis (n = 5)	110L	+	+	0.8	+	+	−	+	−
		120L	+	+	0.9	−	±	−	±	−
		114L	+	+	1.2	+	±	−	±	−
		136L	+	+	1.2	−	+	−	+	−
		98L	+	+	2.6	±	+	±	±	−
	Chronic hepatitis (n = 5)	107L	−	−	0.4	−	−	−	−	−
		127L	−	−	0.6	−	−	−	−	−
		138L	−	−	0.7	−	−	−	−	−
		99L	−	+	1.6	−	−	−	−	−
		100L	−	+	1.7	−	−	−	−	−

+: positive; ±: weakly positive; −: negative.

## Data Availability

Data are continued within the article.
